# Virtual 3D/4D vascular sonobiopsy of the knee joint: validation of the method in the JIA patients treated with ia infliximab or triamcinolone hexacetonide

**DOI:** 10.1186/1546-0096-12-S1-O16

**Published:** 2014-09-17

**Authors:** Miroslav Harjacek, Lovro Lamot, Mandica Vidovic, Marija Perica, Lana Tambic Bukovac

**Affiliations:** 1 Pediatric and Adolescent Rheumatology, Children's Hospital Srebrnjak, Croatia; 2 University of Zagreb School of Medicine, Zagreb, Croatia

## Introduction

In the past few years the use of musculoskeletal ultrasound (MSUS) in pediatric rheumatology has proven to be better than clinical examination in detecting synovitis. Beside standard power doppler ultrasound (PDUS), vascularization of the tissues can be assessed using 3D/4D PD sonography. Using this technique, we can now assess a virtually reconstructed vascular tree within a volume of interest and can ‘objectively’ determine its vascularization by calculating indices using the specially designed VOCAL^TM^ software (Virtual Organ Computer-aided AnaLysis). 3D/4D US is a well established method for determination of accurate vascularity and blood flow in the field of obstetrics and gynecology, but limited data is available in rheumatology patients.

## Objectives

To describe and investigate the ability of 3D/4D PD sonography in accurately predicting vascular and clinical response to IA infliximab (INF) or triamcinolone hexacetonide (TCH) in knee joints of JIA patients.

## Methods

JIA patients (n=15) with clinically active knee synovitis underwent IA injection of either IFX (50 mg/kg; 13 joints in 9 patients) or TCH (1 mg/kg; 8 joints in 6 patients). All patients were treated with first and second line therapy (NSAID, DMARD, corticosteroids). The 3D/4D virtual vascular sonobiopsy was performed by experienced rheumatologists. Patients were examined with the Medison Samsung Accuvix V10 US using a 3D/4D linear probe with 6.2 MHz PD frequency (PRF 1 kHz, gain 45). The VOCAL^TM^ software was utilized to calculate volume of predesigned spheras in the five standardized positions adjusted for the IA size using a specially designed grid. Subsequently, the software automatically displayed sphere volume and three 3D indices: vascularization index (VI), flow index (FI) and vascularization and flow index (VFI). The indices are assumed to reflect the number of vessels within the volume of interest (VI), intensity of flow at the time of 3D sweep (FI), and both blood flow and vascularization (VFI), respectively. Clinical (JADAS) and sonographic (3D/4D US) examination was preformed before and after therapy. To compare indices and analyze correlations, paired samples t-test and Pearson's test were used.

## Results

In total, 21 joints in 15 patients were analyzed using VOCAL software. At the time of IA injections with INF, 13 joints of 9 patients showed grade 3 synovitis in B mode with increased PD signal (2-3/3), while 8 joints of 6 patients treated with TCH showed grade 1-2 synovitis in B mode and increased PD signal (1-2/3). The mean time for follow up was 6.5 months (range 3- 11) for IFX, and 2.8 months (range 1.5-6) for TCH treated patients, respectively. The VI index was expressed as percentage, and both FI and VFI as values of 1-100/cm3. There were significant differences in mean value of VI (p=0,03) and FI/SW (p=0,02) in patients treated with IFX, as well as in JADAS value (p<0,01) in both groups of treated patients. Statistical analysis also showed significant correlations among used indexes.

## Conclusion

3D/4D US appears a valid, sensitive-to-change and feasible method for evaluating knee joint inflammation/vascularization, and possible outcome measure in children with mono/oligoarticular JIA treated with IA TCH, or in selected patients with IA IFX. Further multicenter studies including larger number of patients, other joints and intra and interobserver variability are warranted, before 3D/4D US may be applied as a valid measurement tool suitable for daily clinical practice.

## Disclosure of interest

None declared.

